# Cardiac tamponade mimicking septic shock diagnosed by early echocardiography

**DOI:** 10.4103/0974-2700.66534

**Published:** 2010

**Authors:** Afzal Azim, P B Rao, Piyush Srivastav, Parikshit Singh

**Affiliations:** Department of Critical Care Medicine, Sanjay Gandhi Postgraduate institute of Medical Sciences, Lucknow – 226 014, India

Sir,

Cardiac tamponade is a medical emergency and the overall risk of mortality depends on early diagnosis, treatment, and the underlying cause. Although at first look, a patient presenting with fever, respiratory symptoms, and shock appears to be a case of septic shock of lung origin, this is not universal. An urgent bedside echocardiography is of immense help not only to rule out alternate diagnoses but also to assess the degree of possible myocardial dysfunction in septic shock.

We hereby report a 45-year-old lady presenting with hemodynamic instability and respiratory distress with history of fever and dry cough.

On examination, she was febrile (100°F), respiratory rate of 30–32/min, and cold extremities. Blood pressure (BP) was not recordable. On auscultation, heart rate was 140/min with reduced air entry at lung bases. She was resuscitated and shifted to intensive care unit with a Provisional diagnosis of community-acquired pneumonia with septic shock. Invasive BP was 80/40 mmHg and central venous pressure (CVP) was 16 mmHg with marked respiratory variations. Blood gas showed HCO_3_^-^ of 9.2 mEq/L, central venous saturation of 55% with an arterial lactate of 9 mmol/L. Discontinuing fluids, vasopressors were started. Laboratory parameters were normal except a total leukocyte count of 12,000 and elevated liver enzymes.

Chest radiograph was suggestive of cardiomegaly with bilateral pleural effusion. An urgent transthoracic echocardiography (TTE) revealed pericardial effusion (approx. 1000 mL). Immediate pericardiocentesis was followed by dramatic clinical improvement. Pericardial fluid study revealed hemorrhagic, sterile fluid with malignant cells.

Repeat TTE showed normal left ventricle function with mild pericardial effusion (8 mm lateral to left ventricle—6 mm at apex and 7 mm inferior to right ventricle). Antibiotics were stopped and she was referred to a nearby oncology center where her primaries were found to be ovaries.

Our patient presented with fever, respiratory symptoms, and shock, which prompted us to manage as a case of septic shock of lung origin. But a high CVP, low BP, and cardiomegaly in the chest radiograph with marked respiratory variations was followed by an urgent bedside TTE revealing cardiac tamponade.

Tachycardia and tachypnea were present, but nonspecific and diminished heart sounds and a pericardial rub, which are present in approximately 30% of cases, could not be appreciated.

Pulsus paradoxus was difficult to appreciate in a patient who is restless and tachypneic but could be well visualized in the arterial waveform and pulse oximetry.[1]

Again, she presented with cardiac tamponade with an underlying extracardiac malignancy, which should always be kept as a possibility in sterile fluid collections.[2,3] Although lungs are considered to be the most common source of extracardiac malignancy presenting as cardiac tamponade (58%),[2] ovarian malignancy is also well reported.[4]

This case emphasizes that all cases of fever with hypotension should not be interpreted as septic shock and an early echocardiography must be regarded as a tool for the initial assessment of shock and possible myocardial dysfunction, particularly in septic shock.[5]
